# Driving Behavior Recognition Algorithm Combining Attention Mechanism and Lightweight Network

**DOI:** 10.3390/e24070984

**Published:** 2022-07-16

**Authors:** Lili Wang, Wenjie Yao, Chen Chen, Hailu Yang

**Affiliations:** School of Computer Science and Technology, Harbin University of Science and Technology, Harbin 150080, China; 2020410067@stu.hrbust.edu.cn (W.Y.); chenc@hrbust.edu.cn (C.C.); yanghailu@hrbust.edu.cn (H.Y.)

**Keywords:** driving behavior recognition, feature extraction, attention mechanism, YOLOV4 model

## Abstract

In actual driving scenes, recognizing and preventing drivers’ non-standard driving behavior is helpful in reducing traffic accidents. To resolve the problems of various driving behaviors, a large range of action, and the low recognition accuracy of traditional detection methods, in this paper, a driving behavior recognition algorithm was proposed that combines an attention mechanism and lightweight network. The attention module was integrated into the YOLOV4 model after improving the feature extraction network, and the structure of the attention module was also improved. According to the 20,000 images of the Kaggle dataset, 10 typical driving behaviors were analyzed, processed, and recognized. The comparison and ablation experimental results showed that the fusion of an improved attention mechanism and lightweight network model had good performance in accuracy, model size, and FLOPs.

## 1. Introduction

According to the statistics, most traffic accidents are caused by some interference with normal or non-standard driving behaviors. Among them, playing with mobile phones, making calls, and talking with passengers and other non-standard behaviors account for the majority [1,2]. With the acceleration in urbanization and the increase in per capita income, vehicle ownership is also on the rise. In 2021, the number of motor vehicles in China reached 395 million, of which 302 million were automobiles, and the number of motor vehicle drivers reached 481 million, of which 444 million were motor vehicle drivers. The number of traffic accidents has also increased with vehicle ownership. Therefore, it is significant for traffic safety to recognize non-standard driving behaviors quickly and accurately.

The driving behavior recognition method based on deep learning is considered to be a promising method, which is a practical application. It can be divided into two types: one is classification and recognition based on the traditional convolutional neural network, and the other is object detection and recognition based on the convolutional neural network. Constructed by transfer learning and supervised learning, the convolutional neural network model can recognize driving behaviors such as calling and smoking [3]. Based on the CNN and random decision forest, the driving behavior detection model DriveNet can improve the classification performance [4]. An ensemble model based on the combination of Vgg16 and GoogleNet has been used to identify the driving behavior, which improved the classification accuracy [5]. The feature maps of CNN are fused by convolution kernels of different sizes to realize the recognition of driving behavior by multi-scale network fusion [6]. Improved by regularized pruning, the VGG network can obtain higher accuracy with fewer parameters and greatly save on computing time [7].

The traditional convolutional neural network method can solve the basic problem of driving behavior recognition and classification, but there are still some problems such as less effective feature information and the high similarity between behaviors. Because of the large scale of the network, the amount of computation, and the lack of the real-time performance on the hardware with low performance, the driving behavior recognition algorithm based on the convolutional neural network still has great problems in practical application. In contrast, an object detection and recognition algorithm based on a convolutional neural network has strong robustness and adapt ability, it improves the detection accuracy and real-time speed significantly, and reduces the parameter quantity and floating point computation.

The two-stage object detection algorithm is based on candidate regions. Based on the fusion model of DRN and Faster R-CNN, a behavior recognition algorithm replaces two-layer residual blocks with three-layer dilated convolution residual blocks, which has achieved a better recognition effect in the behavior recognition. However, due to the large size of the model, the real-time performance is obviously insufficient [8]. Based on MobileNetV3 and ST-SRU, an algorithm was used to recognize dangerous driving behaviors. It estimates the two-dimensional coordinates of the joints and classifies the actions according to the skeleton sequences of the actions. Its accuracy is better with fewer parameters, and the real-time is improved, however, the model only obtains good performance in a simulated driving environment, and the generalization ability is not strong [9]. Based on the Tutor–Student network, the driving behavior recognition algorithm divides the driving behaviors into two sub-tasks: action localization and action classification. After guidance by the tutor network, the student model has high recognition accuracy and strong robustness, but the computation expense is too high for low-performance devices [10]. Based on the improved SSD, the driving behavior recognition algorithm uses the residual learning to make the network learning easier, and introduces a multi-layer feature pyramid to improve the object detection accuracy, but the recognition accuracy changes greatly with different detection environments, and the generalization ability is insufficient [11].

To improve the driving behavior detection accuracy and detection speed, for the issues of a large number of parameters, less effective feature information, and low detection speed, in this paper, a fusion driving behavior recognition algorithm with an attention mechanism and a lightweight network is proposed. The algorithm selects YOLOV4 as the basic framework. For the lightweight network parameters, the YOLOV4 [12] feature extraction network was reconstructed with the lightweight network MobileNetV3 [13], and the 3×3 convolutions in the FPN network was replaced by 1×1 convolution. To retain the effective information of the driving behaviors and reduce the influence of useless information, improved channel attention mechanism and spatial attention mechanism were introduced. To verify the effect of the lightweight network and attention mechanism on the network, ablation experiments were carried out. The results show that the algorithm maintained a high behavior recognition accuracy with the reduction in the parameters. Compared with the current mainstream object detection algorithms, the algorithm in this paper still had good performance.

## 2. Algorithm Principle

### 2.1. MobileNetV3 Network

MobileNetV3 introduces depth-wise separable convolution as an effective alternative to traditional convolution layers, and it uses linear bottlenecks and inverted residual structures to produce more efficient layer structures by simplifying the difficulty of the problem [14,15]. Depth-wise separable convolution effectively decomposes traditional convolution by separating spatial filtering from the feature generation mechanism. The depth-wise separable convolution is defined by two separate layers: the lightweight depth-wise convolution for spatial filtering and the heavier 1×1 pointwise convolution for feature generation.

Depth-wise convolution is different from conventional convolution operations. In depth-wise convolution, one convolution kernel has only one dimension, which is responsible for each channel, and one channel is convolved by only one convolution kernel. In conventional convolution, the dimension of each convolution kernel is the same as the input dimension, and each channel is added after separate convolution operation [16].

**Figure 1:**
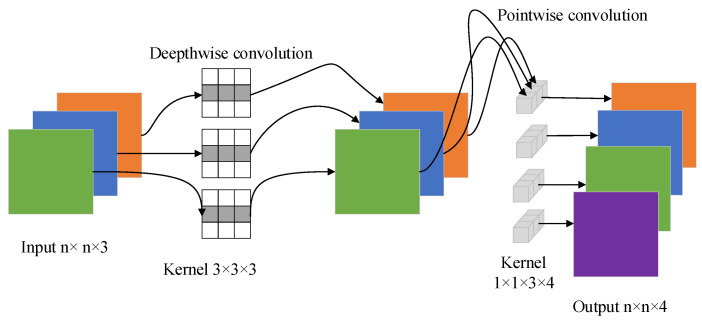
The depth-wise separable convolution.

After depth-wise convolution, the number of channels in the output feature map is the same as that in the input layer, and the number of channels cannot be increased. Moreover, this operation carries out an independent convolution operation for each channel of the input layer, and it cannot effectively utilize the characteristic information of different channels in the same spatial position. Therefore, pointwise convolution is required to combine the generated feature images to generate new feature images. The structure of the depth-wise separable convolution network is shown in Figure 1.

**Figure 2:**
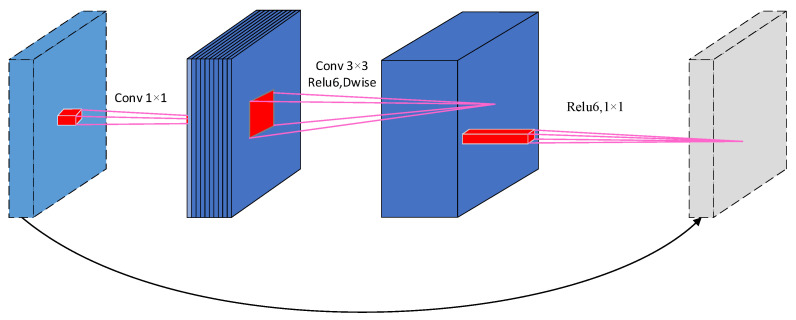
The linear bottleneck and inverted residual structure.

The linear bottleneck and inverted residual structures are defined by depth-wise convolution and 1×1 projection layers after 1×1 extended convolution. The input and output are connected to the remaining connections only if they have the same number of channels. This structure maintains a compact representation at the input and output, while it extends internally into higher-dimensional feature spaces, which can increase the expressiveness of the nonlinear transformation of each channel. The linear bottleneck and inverted residual structure are shown in Figure 2.

### 2.2. Attention Mechanism

**Figure 3:**
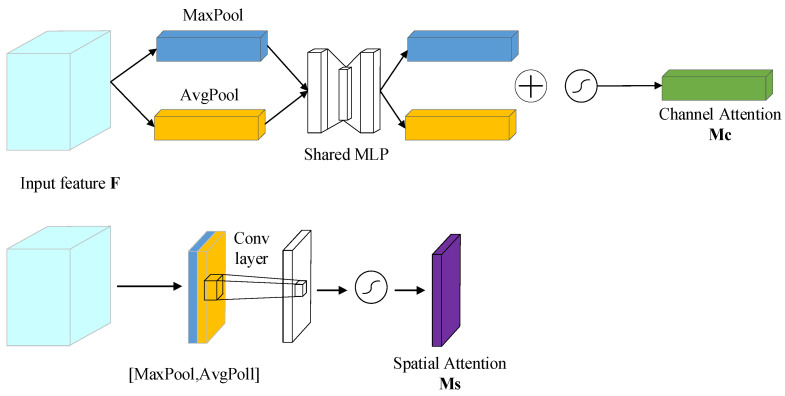
The process of attention realization.

The visual attention mechanism is a special brain signal processing mechanism in human vision [17]. It captures the object area by scanning the image quickly, and pays more attention to obtaining detailed information and suppresses other useless information. For humans, it is a way to quickly sift through a lot of information with limited attention. In the existing semantic segmentation system, the pyramid structure can extract the feature information of different scales, but it lacks the priority attention of a global context. Therefore, using the attention mechanism to add new connections to the traditional neural network, it is possible to automatically determine how much attention needs to be allocated to each part of the input. Therefore, accurate pixel-level attention can be provided to the features extracted by the convolutional neural network. The channel attention mechanism (CAM) and spatial attention mechanism (SAM) are two commonly used attention mechanisms in convolutional neural networks. The channel attention mechanism is a one-dimensional feature map in which each channel is assigned a weight. The spatial attention mechanism assigns a weight to each pixel in the feature map, which is a two-dimensional feature map. The process of attention realization is shown in Figure 3, and the algorithm is described as Equations (1) and (2).

(1) $$F^{\prime }=M_{c}\left(F\right)⊗F,$$

(2) $$F^{\prime \prime }=M_{s}\left(F^{\prime }\right)⊗F^{\prime },$$

In Equations (1) and (2), *F* is the input image tensor, *H* is the length of the image, *W* is the width of the image, and C is the number of channels. $M_{c}\left(F\right)$ is the channel attention, $F^{\prime }$ is the adjusted output of the channel attention, $M_{s}\left(F^{\prime }\right)$ is the spatial attention, and $F^{\prime \prime }$ is the adjusted output of the spatial attention.

## 3. Algorithm Improvement

After adopting many optimization strategies to improve its own shortcomings, the YOLOv4 object detection algorithm performed well in various evaluation indexes under the standard dataset of high-performance devices. When the algorithm is deployed on mobile devices with poor hardware performance, it does not need high accuracy, but high prediction speed, according to different application environments. In the case of limited computing power and memory resources of mobile devices, the size of the algorithm model becomes particularly important. Obviously, the YOLOv4 algorithm is difficult to apply to mobile object detection devices. Therefore, in this paper, an improved object detection algorithm based onYOLOv4 is proposed, and there are two innovations as follows:

1. The feature extraction network of YOLOV4 is improved. The model is pruned and the parameter quantity is reduced, but the accuracy of the network is not reduced. The CSPDarknet53 in YOLOV4 is replaced by the improved MObileNetV3 network model.
2. The attention mechanism structure is improved, the weights of the invalid features are reduced to retain effective features and improve the identification accuracy of the driving behavior.

**Figure 4:**
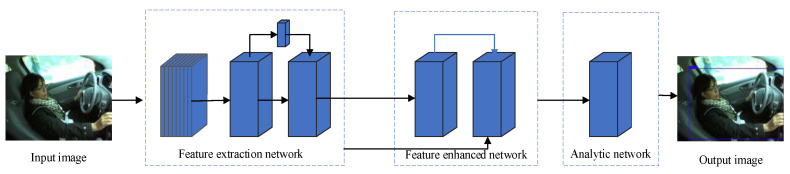
The detection network of the driving behaviors.

Figure 4 shows the driving behavior detection model built in this paper, which is mainly composed of a feature extraction network and a driving behavior detection network. The input image data obtained high-level semantic features through the feature extraction network, and the features were fused through the attention mechanism. Afterward, the detection network predicts the position and size of the driving behavior and obtains the prediction boundary box.

### 3.1. Improvement of Feature Extraction Network

The feature extraction network of the driving behavior recognition model was constructed based on MobileNetV3 and integrated with the attention mechanism. In order to use the location information of the shallow feature image and semantic information of the deep feature image, the feature fusion of shallow feature image and deep feature image wascarried out in the attention network. This was implemented inthe following steps: first, the number of feature parameters was reduced by deep separable convolution operation, and then the feature images were expanded and input into the attention module after up-sampling, and finally, three different feature images were generated.

In this paper, 640 × 480 images were created as three-channel images and input into the network. The original image size was 416 × 416, and after five operations of the bottleneck structure in MobileNetV3, three effective feature layers were obtained, and their sizes were 52 × 52, 26 × 26, and 13 × 13, respectively. The 13 × 13 feature layers were input into the spatial pyramid pooling (SPP) network, and feature fusion was carried out by different sizes pooling layers to improve the receptive field and separate effective features.

Then, the three groups of feature layers were input into the path aggregation network (PANet) for fusion. The bottom-up feature fusion path in PANet can effectively integrate richer feature information. To further reduce the number of network parameters, the 3×3 traditional convolutions in PANet were replaced by depth-wise separable convolution.

Finally, the three groups of feature layers after feature fusion predict the three boundary boxes for each position. There were 10 types of driving actions in the dataset. During identification and prediction, the network generated (5 + 10) predictive values for each boundary box, among which the first four values were abscissa, ordinate, width of the prediction box, and height of the prediction box. The fifth value was the confidence degree that the object is predicted as a certain category, and the following values were the 10 predicted category labels.

### 3.2. Improvement of Attention Mechanism

In the process of feature extraction by the convolutional network, with the increase in the network depth, the size of the feature map continues to decrease, and the number of channels continues to increase. There are more outline features in the low-level network, and each feature map in the high-level network has rich semantic information. Different feature maps only contain a part of the semantic feature information of the driving behavior. At the same network level, the semantic information of different channel features is combined into the representation of driving behaviors in the current network layer [18]. The expression of driving behavior in a feature map can be defined as Equation (3):

(3) $$occl\left(n\right)=\left[v_{0}p_{0},v_{1}p_{1},\ldots ,v_{k}p_{k}\right],$$

In Equation (3), n is the *n*-th feature map in a convolutional layer, $p_{i}$ is the *i*-th region of a feature layer, $v_{i}\in \left\{0,1\right\}$, i∈[0,k], $v_{i}p_{i}$ represents whether there is driving behavior information in the *i*-th region.

Because the weight of the traditional CNN channel is usually fixed and equal, the ability for a different expression of the network is limited. If the weight of each channel is recalculated, the feature channel of the object’s visible region contributes more to the final convolution feature, which can highlight the object feature in the background. The weight of the channel can be calculated as Equation (4):

(4) $$F_{occl}\left(n\right)=\omega_{n}F_{c},$$

In Equation (4), $F_{c}$ is the feature channel, and $\omega_{n}$ is the weight. The channel attention mechanism is to continuously learn new $\omega_{n}$ and reweigh the channel, so that the network can adapt to different feature channels.

In MobileNetV3, it only uses the channel attention mechanism and ignores the importance of spatial information for the feature map. The spatial information of feature maps is helpful to the network to focus on the object’s regions of interest, so the feature channel attention mechanism and spatial attention mechanism can be used in the image description [19]. When the spatial attention mechanism is applied to the object detection, useful features are highlighted in the network [20]. The feature spatial information was used in the driving behavior detection, and a spatial attention module was constructed to highlight the driver object region. Based on the spatial information of the feature map, the spatial attention module obtains the weights of the spatial attention and reactivates the input features to lead the network to pay attention to the driving behavior and suppress the background interference.

The attention network consists of two sub-modules: channel attention and space attention. In the attention network, the input features are connected in channel dimensions, $F\in R^{H*W*C}$*F*, and then *F* is input into the channel attention and spatial attention module for feature fusion.

From the above analysis, according to the attention model, the channel information and the spatial information of the feature map can be constructed, and the network can strengthen the presentation ability of the regional features and obtain the position of the region of interest. It uses the effective features and suppresses the useless information. To improve the accuracy of the detection of continuous actions, the residuals with dilatative convolution were used to reduce the parameter quantity in the spatial attention model, and the sensing field was also improved.

#### 3.2.1. Improvement of Channel Attention

Channel attention focuses on the input feature map. First, global maximum pooling and mean pooling are used to map the feature information to form two different channel descriptions.$F_{avg}^{c}$ represents the channel information after average pooling for *F*, and $F_{max}^{c}$ represents the channel information after maximum pooling *F*.

Because of the low computational budget, lightweight convolutional neural networks are limited in the depth and width of CNNs, which led to the decline in the model performance and the limitation in representation ability.

**Figure 5:**
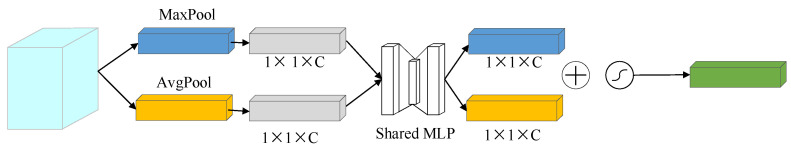
The improved channel attention structure.

In this paper, a one-dimensional convolution with adaptive dimension *k* was adapted to aggregate the feature information of k neighborhood channels. The size of the convolution kernel can be adaptively adjusted according to the number of channels. The information of the two channels were added together and activated by sigmoid function to generate channel attention $M_{c}\left(F\right)\in R^{C*1*1}$, and were then multiplied with the original input feature to inject the channel attention mechanism. The specific calculation process is shown in Equations (5) and (6). The attention structure of the improved feature channel is shown in Figure 5.

(5) $$M_{c}\left(F\right)=\sigma (f_{1d}^{k}\left(AvgPool\left(F\right)+f_{1d}^{k}\left(MaxPool\left(F\right)\right)\right)=\sigma \left(f_{1d}^{k}\left(F_{avg}^{c}\right)+f_{1d}^{k}\left(F_{max}^{c}\right)\right),$$

In Equation (5), *σ* represents sigmoid activation function, and $f_{1d}^{k}$ represents a one-dimensional convolution operation with convolution whose kernel size is *k*.

(6) $$k={\left|\frac{log_{2}\left(C\right)}{2}+\frac{1}{2}\right|}_{odd}$$

In Equation (6), *C* is the number of channel of the feature map, ${\left|*\right|}_{odd}$ is the nearest odd number to *, and ${\left|*\right|}_{odd}\leq *$.

#### 3.2.2. Improvement of Spatial Attention Module

**Figure 6:**
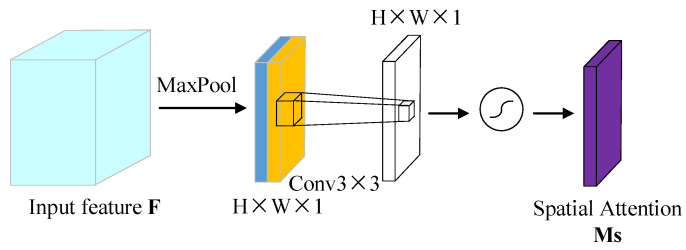
The improved spatial attention structure.

Because the useful information of the detected object is usually covered by the background, when the feature expression is enhanced through the channel attention module, the spatial location of the useful information needs to be determined. Unlike the channel attention mechanism, the spatial attention mechanism is mainly used to highlight the region associated with the current task in the feature map, which is to guide the network to focus on the visible region of the object. To solve the problem of network degradation caused by the addition of the convolution layer in a deep network, the convolution structure in the original network is replaced by the residual structure with dilated convolution. As shown in Figure 6, in the spatial attention module, the channel attention was introduced into feature information, and global average pooling (GAP) and global max pooling (GMP) were carried out. Two different types of channel information $F_{avg}^{\prime c}$ and $F_{max}^{\prime c}$ were generated and concatenated to generate a more effective spatial feature layer. Then, the residual structure with dilatative convolution was used to further aggregate the information in the upper and lower space to improve the receptive field. After sigmoid function activation, the spatial attention model $M_{s}\left(F\right)\in R^{1*H*W}$ was generated. Finally, the spatial attention model $M_{s}\left(F\right)$ was multiplied by the corresponding elements of the input feature to inject the spatial attention mechanism.

The specific calculation process is shown in Equation (7):

(7) $$M_{s}\left(F\right)=\sigma \left(\left(1+f_{dilation}^{3*3}f_{2d}^{1*1}\right)\left(GAP\left(F\right)+GMP\left(F\right)\right)\right)=\sigma \left(\left(1+f_{dilation}^{3*3}f_{2d}^{1*1}\right)\left(F_{avg}^{\prime c}+F_{max}^{\prime c}\right)\right),$$

In Equation (7), $f_{dilation}^{3*3}$ represents the expansion convolution with the convolution kernel size of 3, and $f_{2d}^{1*1}$ represents the standard convolution whose kernel size is1.

### 3.3. Improvement of Loss Function

The driving behavior detection task was regarded as a kind of high-level semantic feature detection. On the basis of the semantic features, the final prediction boundary box was obtained through the driving behavior parsing network. In this paper, the driver’s position, category, and height were predicted, and the boundary box was obtained by simple geometric conversion. After obtaining the driver’s predicted height *h*, according to the ratio of the height to the width *a*, the width of the boundary box w=h∗a can be calculated.

When the lightweight YOLOV4 detects the driving behavior, it first determines the position of the object in the annotated image, and then classifies the object in the ground truth box. It can be described as follows: input the image *X*, locate and classify the image according to the task requirements, and adjust the loss of anchor box $L_{conf}$ and confidence $L_{loc}$. The loss function is shown in Equation (8).

(8) $$Lx,c,l,g=\frac{1}{N}\left(L_{conf}\left(x,c\right)+\partial L_{loc}\left(x,l,g\right)\right),$$

In Equation (8), *N* is the number of anchor box, ∂ is the scale between $L_{conf}$ and $L_{loc}$, whose default value is 1; *c* is the predicted value of the category confidence; *l* is the anchor box position of the boundary box; *g* is the position parameter of the real object; *x* is an indicator parameter whose standard form is $x_{ij}^{p}\in \left\{1,0\right\}$, which is the probability of *p* class when the *i*-th anchor box matches the *j*-th object.

The position loss function $L_{loc}$ adopts smoothL1. It combines the advantages of L1 loss and L2 loss, which can speed up network training and smoothen the gradient of the object image changes. The formula is shown as Equation (9), and the parameters in Equation (9) are shown in Equations (10)–(14).

(9) $$L_{loc}\left(x,l,g\right)=\overset{N}{\underset{i\in Pos}{\sum }}\underset{m\in \left\{cx,cy,w,h\right\}}{\sum }x_{ij}^{k}smooth_{L1}\left(l_{i}^{m}-{\hat{g}}_{j}^{m}\right),$$

(10) $${\hat{g}}_{j}^{cx}=\frac{g_{j}^{cx}-d_{i}^{cx}}{d_{i}^{w}},$$

(11) $${\hat{g}}_{j}^{cy}=\frac{g_{j}^{cy}-d_{i}^{cy}}{d_{i}^{h}},$$

(12) $${\hat{g}}_{j}^{w}=\log\left(\frac{g_{j}^{w}}{d_{i}^{w}}\right),$$

(13) $${\hat{g}}_{j}^{h}=\log\left(\frac{g_{j}^{h}}{d_{i}^{h}}\right),$$

(14) $$smooth_{L1}\left(x\right)=\{\begin{array}{c} 0.5x^{2}if\left|x\right|<1\\ \left|x\right|-0.5\;\;\;otherwise \end{array},$$

According to $x_{ij}^{p}$, only positive samples work in training the anchor box, therefore, the SoftMax loss function is used for the probability loss of the category, which is composed of the SoftMax and cross entropy loss. The loss function becomes smaller when the predicted value is closer to the true value and vice versa. The optimization process increasingly showed more predicted values close to the true values, thus reducing the loss function to speed up the fitting, which is shown in Equation (15):

(15) $$L_{conf}\left(x,c\right)=-\overset{N}{\underset{i\in Pos}{\sum }}x_{ij}^{p}\log\left({\hat{c}}_{i}^{p}\right)-\underset{i\in neg}{\sum }\log({\hat{c}}_{i}^{0}),$$

In Equation (15), ${\hat{c}}_{i}^{p}$ represents the probability that the *i*-th anchor box is predicted as *p*, and ${\hat{c}}_{i}^{0}$ represents the probability that the *i*-th category is predicted as the foreground when *p* is predicted.

## 4. Experiment and Analysis

### 4.1. Experimental Settings

#### 4.1.1. Experimental Environment

The hardware configuration of the experimental platform used for training wasa workstation configured with an AMD EPYC 7543 processor, a main frequency of 2.00 GHz, NVIDIA RTX A5000 GPU, 24 G memory, and the OS wasUbuntu18.

#### 4.1.2. Dataset

**Figure 7:**
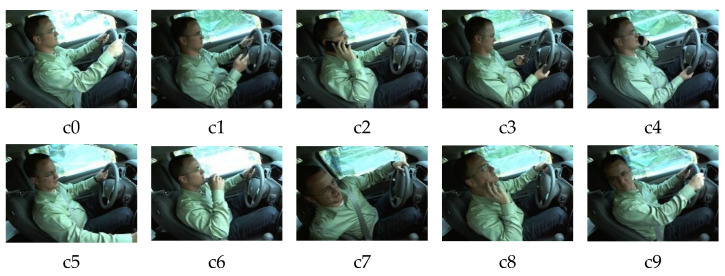
Distracted driving behaviors.

To apply the driving behavior recognition method, a real environment dataset was selected. In this paper, the dataset was composed of 20,340 images, which were provided by Kaggle, and a total of 8000 images were selected as the validation set to test the generalization ability of the model. The dataset contained 10 categories:c0 (normal driving), c1 (a mobile phone in the right hand), c2 (a mobile phone in the left hand), c3 (making a phone call with the right hand), c4 (making a phone call with the left hand), c5 (operating media devices), c6 (taking items from the backseat), c7 (making up), c8 (drinking), and c9 (talking with passengers), which are shown in Figure 7. Each category contains 2034 images, which were annotated with XML according to the format of the YOLO algorithm, and the training set and the validation set were randomly divided according to the ratio of 9:1.

#### 4.1.3. Data Preprocessing

The distracted driving behaviors were annotated by the labelImg, and XML files were generated corresponding for all of the images including length and width, category of the ground truth box, lower left coordinates (xmin, ymin), and upper right coordinates (xmax, ymax) of the ground truth box. The process of the image annotation is as follows:

1. Distracted driving behaviors are mainly upper body movements including head movements, hand movements, and where the hands are put on the steering wheel.
2. When annotating the images, the anchor box is mainly limited from the area of the driver’s head to the legs and the back to the steering wheel to avoid unnecessary space.
3. After annotating the anchor box, the size of the box can be obtained, and the image size and anchor box are normalized, which were input into the model.

#### 4.1.4. Evaluation Indexes

In this paper, the mean average precision (mAP), the model parameter quantity (params), and floating-point operations (FLOPs) were used to evaluate the quality of the model algorithm, and the statistical significance test (*t*-test) was used in the ablation experiment to prove that it was superior to the other models.

The calculation formulas of recall, precision, and mAP are shown in Equations (16)–(18). TP is the positive sample that is correctly judged, FP is the positive sample that is incorrectly predicted, FN is the negative sample that is incorrectly judged, and TN is the negative sample that is correctly judged. Recall represents the proportion of correctly judged positive samples in all of the correctly judged samples, and precision represents the proportion of the positive samples correctly judged in all of the judged positive samples. AP is the area surrounded by the curve drawn with recall as the *X*-axis and precision as the *Y*-axis, and mAP is the mean of the AP values of all samples. Ten driving behaviors were recognized in this experiment.

(16) $$Recall=\frac{TP}{TP+FN},$$

(17) $$Precision=\frac{TP}{TP+FP},$$

(18) $$mAP=\frac{1}{C}\underset{c\in C}{\sum }AP\left(c\right),$$

The model parameter quantity determines the size of the model files and also fixes the memory usage during the inference of the model. The calculation is shown in Equation (19):

(19) $$Parameters=k_{t}\times k_{w}\times k_{h}\times c_{i}\times c_{0}+c_{0},$$

FLOPs refer to the calculation for the inference of the model, and the calculation is shown in Equation (20):

(20) $$FLOPs=k_{t}\times k_{w}\times k_{h}\times t\times w\times h\times c_{i}\times c_{0},$$

In Equations (19) and (20), $k_{t}$ is the convolution time; $k_{w}$ is the convolution kernel width; $k_{h}$ is the height of convolution kernel; t  is the input time of the feature map; w is the width of feature map; h is the height of feature map; $c_{i}$ is the number of input feature maps; and $c_{0}$ is the number of output feature maps.

#### 4.1.5. Training and Model Parameters

In the training phase, the backbone network was frozen in the first 50 epochs, and all of the network parameters can be updated in the last 50 epochs. The maximum learning rate was set to 0.001, and the cosine annealing was used to adjust the learning rate. The minimum learning rate was 10^−5^, the batch size was 64, and the Adam algorithm was adopted to optimize the network parameters.

For the statistical test of significance, on the validation set, the mAP was calculated every five training rounds.

### 4.2. Experimental Results

#### 4.2.1. Ablation Experiments

In this paper, the real-time detection of a driving behavior recognition algorithm was implemented. Based on YOLOV4 as the basic network, its feature extraction module is lightweight and an attention mechanism was added. To verify the effectiveness of the proposed algorithm with the same training dataset, ablation experiments were carried out on YOLOV4, YOLOV4 with a lightweight feature extraction, YOLOV4 with an attention mechanism, YOLOV4 with a lightweight extraction network and attention mechanism separately. The evaluations of mAP, the parameter quantity, and FLOPs of each model were compared, which are shown in Table 1.

The above results show that the performance of the algorithm in this paper was greatly improved after the lightweight transformation and the addition of the attention mechanism. When comparing YOLOV4 with YOLOV4 + MobileNetV3, YOLOV4 paid more attention to extracting the feature layer, and obtained the optimal model to improve the accuracy with the same training parameters. YOLOV4 + MobileNetV3 paid more attention to the importance of different features in the channel dimension and the spatial dimension, and obtained better accuracy with the same condition of parameter quantity and FLOPs. These mean that both the lightweight and attention mechanism play a positive role in model optimization. Therefore, after fusing the above methods, the network pays attention to the key information of space and channel, and maintains a low amount of computation and parameter quantity.

**Figure 8:**
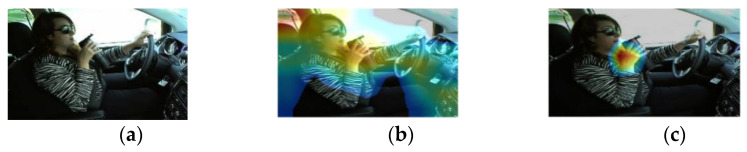
Heat map. (**a**) Original image; (**b**) Heap map byYOLOV4 + MobileNetV3; (**c**) Heap map byour algorithm.

To acquaint the influence of the attention module on the performance of the detector, the visualized activation diagram of the position prediction $H_{center}$ is presented in Figure 8. Figure 8a shows an original image, 8b and 8c are the heap maps processed by YOLOV4 + MobileNetV3 and our algorithm, respectively. From Figure 8, it can be seen that 8c was recognized closer to the motion region, while in 8b, there were still background interferences. This proves that the attentional mechanism directs the network to focus on the recognized region and reduces the interferences of the background noise.

**Figure 9:**
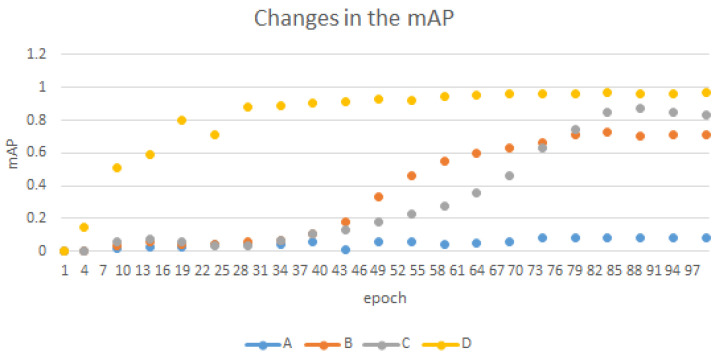
The scatter plot of the mAP changes during training.

In the training process of the above four algorithms, each algorithm calculated 20 mAP values. The two-sided *t*-test was used for the statistical significance test. The mAP changes are shown in Figure 9.

When the statistical significance level α < 0.05, it was regarded as reaching the significance level, which is shown in Table 2:

From Table 2 and Figure 9, our algorithm showed excellent performance in both the statistical significance indicators and mAP during training.

#### 4.2.2. Comparative Experiment

After the ablation experiment above mentioned, our algorithm had the highest performance. To further verify the quality, the algorithm was compared with the current mainstream driving behavior recognition algorithms, and the results are shown in Table 3.

According to Table 3, the recognition accuracy of Drive-Net was the highest, but it could only be used for the image classification of driving behavior, and it did not have real-time performance. The ST-SRU driving behavior recognition algorithm had higher accuracy and fewer parameters and floating-point operations, but the experiment was carried out in their own simulated driving behavior dataset, and the performance was poor in the real environment. The Tutor–Student driving behavior recognition algorithm had a high performance, accuracy, and low calculation amount, but its model parameter quantity is too large to deploy. The SSD driving behavior recognition algorithm had a high accuracy rate, but its model parameter quantity and floating-point calculation amount were the highest, which is not suitable for lower-level equipment. The LSTM algorithm has a simple structure, and its parameter quantity and floating-point operations were the lowest. However, the input images were infrared extraction, and all of the features of the images need to be analyzed during inference, so there is insufficient feature information, and the recognition accuracy was the lowest. Our algorithm uses the idea of object detection and has real-time performance. It implements feature extraction and network lightweight processing on YOLOV4, and has a low parameter quantity as well as calculations with high accuracy. With the attention mechanism, it guides the network to focus on the channel and the spatial information to improve the detection effect.

## 5. Conclusions

In this paper, YOLOV4 and MobileNetV3 were fused, the model parameter quantity was further reduced by using the lightweight deep separable convolution, and the channel attention and spatial attention were improved. On the Kaggle dataset, the accuracy of our algorithm achieved 96.49%, the parameters of the model occupied 12.629 M, and the FLOPs was10.652 G. It can also be used for real-time detection. However, in the practical application of the driving behavior recognition algorithm, various factors such as continuity, diversity, and coincidence of driver actions should be taken into account. A time sequence network can be introduced to perform the time sequence analysis of actions, or fuse multi-feature network to prevent false detection caused by a single detection method. There is still some improvements to be made in detection and tracking in fast movement scenes, and the real-time performance will decline in deeper networks. In the future, we will devote study as to how to prune the model to further simplify the network structure to meet the actual deployment applications.

## Figures and Tables

**Table 1:** Results of the ablation experiment.

Model	mAP/%	Params/M	FLOPs/G
YOLOV4	7.93	64.363	60.527
YOLOV4 + MobileNetV3	80.5	40.692	39.652
YOLOV4 + SA + CA	80.4	64.363	60.527
Our algorithm	96.49	12.629	10.652

**Table 2:** The *t*-test.

Significant Level	YOLOV4 & Our Algorithm	YOLOV4 + MobileNetV3 & Our Algorithm	YOLOV4 + SA + CA & Our Algorithm
α	2 × 10^−15^	9.75 × 10^−6^	8.63 × 10^−6^

**Table 3:** The results of the different algorithms.

Model	mAP/%	Params/M	FLOPs/G
Drive-Net [4]	95	-	-
ST-SRU [9]	95.6	2.863	7.42
Tutor–Student [10]	96.29	34.71	11.4
SSD [11]	94.65	26.285	119.131
LSTM [21]	88.15	**1.863**	**1.995**
Our algorithm	**96.49**	12.629	10.652

## Data Availability

The data that support the findings of this study are openly available in Kaggle at https://www.kaggle.com/c/state-farm-distracted-driver-detection/data.
